# Successful pharmaco-mechanical treatment of a subtotally occluded venous bypass graft in a patient presenting with acute coronary syndrome: a case report and review of the current literature on the role of local thrombolysis

**DOI:** 10.3389/fcvm.2025.1471462

**Published:** 2025-03-17

**Authors:** Matthias Renker, Samuel Sossalla, Christoph Schoefthaler, Grigorios Korosoglou

**Affiliations:** ^1^ Department of Cardiology, Campus Kerckhoff of the Justus-Liebig-University Giessen, Bad Nauheim, Germany; ^2^ German Centre for Cardiovascular Research (DZHK), Partner Site RheinMain, Frankfurt am Main, Germany; ^3^ Department of Cardiology and Angiology, Justus-Liebig-University Giessen, Giessen, Germany; ^4^ Department of Cardiology and Vascular Medicine, GRN Hospital Weinheim, Weinheim, Germany; ^5^ Cardiac Imaging Center Weinheim, Hector Foundation, Weinheim, Germany

**Keywords:** acute coronary syndrome, computed tomography angiography, coronary artery bypass graft, percutaneous coronary intervention, thrombectomy, local thrombolysis

## Abstract

Coronary artery bypass grafting (CABG) is a common and effective treatment for patients with complex coronary artery disease. This case report discusses a 75-year-old male patient who presented with angina and shortness of breath due to thrombus formation in a venous graft 20 years after CABG. Initial diagnostics indicated non-ST-elevation myocardial infarction, leading to immediate intervention. Cardiac catheterization revealed thrombus in the vein graft to the large first diagonal branch, necessitating percutaneous coronary intervention. Despite initial efforts, thrombus aspiration and further catheter advancement were unsuccessful. A combination of balloon angioplasty, stent implantation, and intra-arterial thrombolysis with recombinant tissue plasminogen activator (rt-PA) was employed, resulting in significant thrombus reduction and improved coronary flow. Follow-up coronary CT angiography (CCTA) confirmed complete thrombus resolution and patent graft. The patient was discharged with dual antiplatelet therapy and showed favorable outcomes. This case emphasizes the challenges of managing thrombotic complications in venous bypass grafts and highlights the effectiveness of a multifaceted interventional approach combined with CCTA for non-invasive patient follow-up and assessment of treatment success. Furthermore, a review of the current literature on the role of local thrombolysis for occluded coronary artery bypass grafts is provided.

## Introduction

Coronary artery bypass grafting (CABG) is the most frequently performed procedure in cardiac surgery (1), being the foremost preferred strategy of myocardial revascularization in patients with extensive, multi-vessel coronary artery disease (CAD). Beyond improvements in cardiovascular outcomes (2), patients undergoing CABG were reported to exhibit low clinical event rates and improved quality of life in recent prospective cohort studies (3).

Long-term patient outcomes after CABG are primarily dependent on the patency of grafts used for revascularization (1, 4). The incidence of graft failure is reported to be in the range of 10%–50% within approximately 10 years, with the highest failure rates found for venous graft material (1, 4, 5). Graft failure can occur within 1 month of surgery or later with different underlying causes. Most often, neointimal hyperplasia leads to obstructing atherosclerotic plaque formation being the primary cause of late vein graft failure, which may be associated with superimposed thrombus (5). Treatment of failed venous grafts—whether surgical or interventional—is feasible, but carries a high risk of peri-procedural complications. Furthermore, it is unclear whether in case of venous graft failure, the treatment of the native recipient vessel could be safer and more effective compared to the treatment of the occluded graft (6, 7).

Herein, we present the case of a 75-year-old man with recurrent ischemia due to thrombus formation in a venous bypass graft.

## Case description

**Figure 1:**
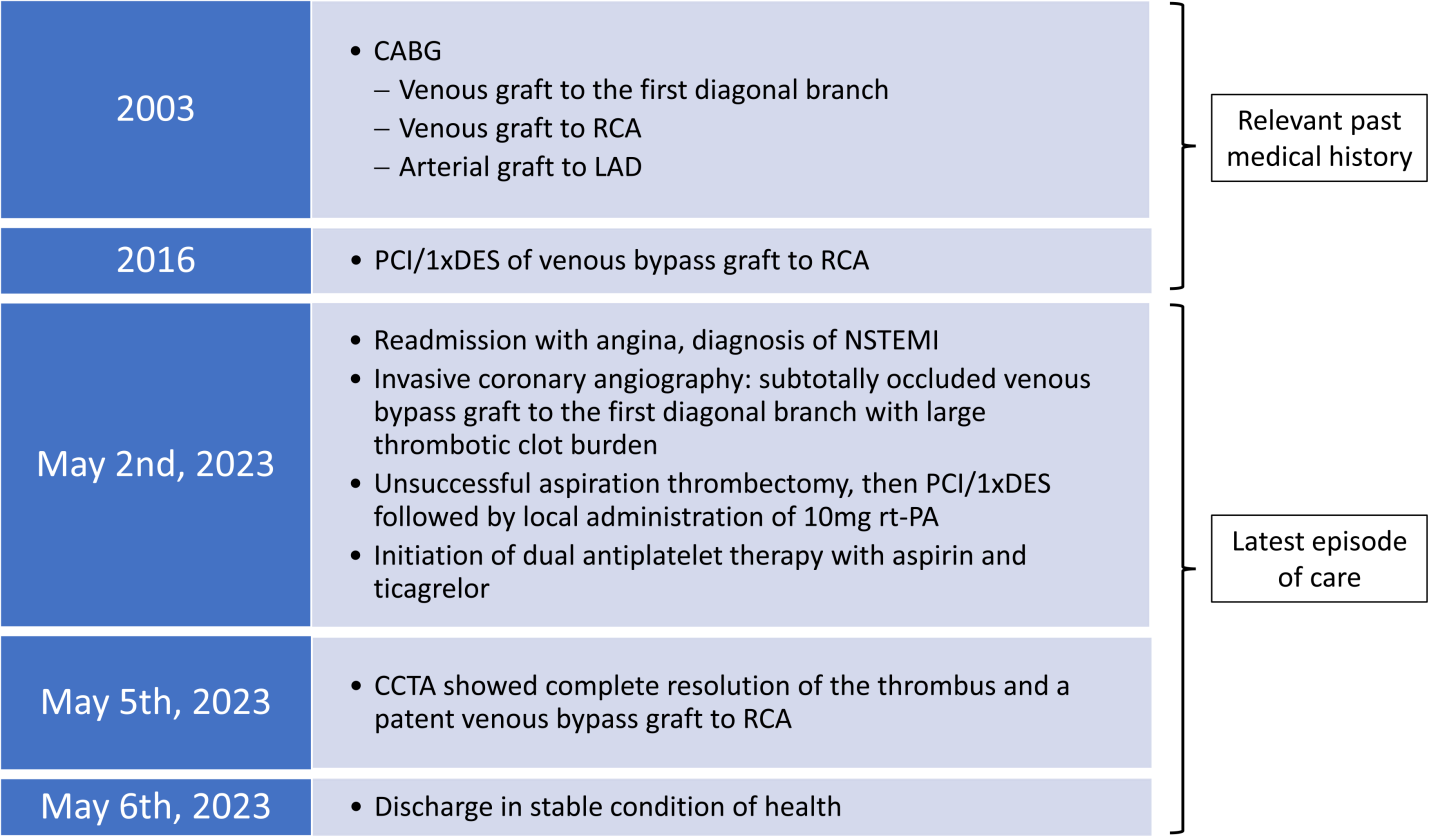
Chronological presentation of relevant data from the patient's cardiac medical history and the latest episode of care.

A 75-year-old male patient presented to our emergency department with suspected acute coronary syndrome (ACS) due to new onset angina and shortness of breath (case flowchart in Figure 1). He had undergone CABG 20 years ago and percutaneous coronary intervention (PCI) with placement of one drug-eluting stent (DES) into the vein graft to his right coronary artery (RCA) 7 years ago at our department. Beyond a history of hypertension, hyperlipidemia, and type 2 diabetes mellitus, no other clinical health conditions were known.

The physical examination exhibited an elevated heart rate (100 bpm), which electrocardiogram confirmed to be sinus tachycardia without ischemic signs. Echocardiography revealed mildly reduced systolic left ventricular function without regional wall motion abnormalities. Because laboratory chemical diagnostics revealed an elevated and increasing high-sensitive cardiac troponin T (84.8–165.7 ng/L at 2 h), the diagnosis of non-ST-elevation myocardial infarction (NSTEMI) was established. In addition, chronic renal failure was present with a creatinine value of 1.86 mg/dl. Subsequently, the patient received aspirin i.v., the loading dose of ticagrelor p.o., and fondaparinux s.c. and was transferred to the intensive care unit.

**Figure 2:**
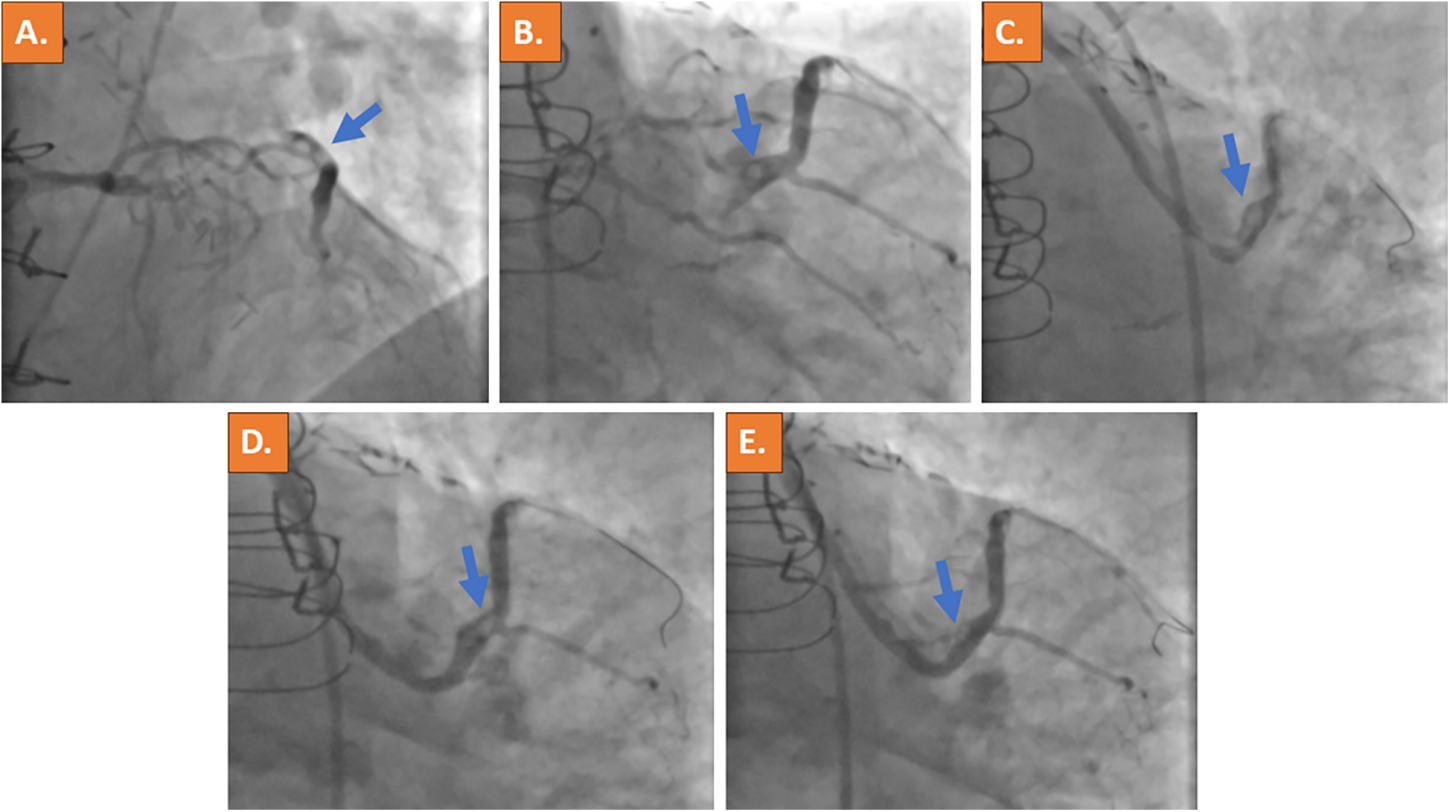
Acute invasive coronary angiography with selective contrast material injection into the left coronary artery ostium revealed retrograde filling of the vein bypass graft to the large first diagonal branch with suspected thrombus formation at the distal site of the bypass anastomosis (blue arrows in **A,B**). A high-grade stenosis of the vein graft was present **(C)**. After PCI, a remaining thrombus was noticed distally to the implanted DESs **(D)**. Because thrombus material remained, intra-arterial application of thrombolysis (rt-PA) was performed and significantly reduced the clot burden **(E)**. The thrombotic material is indicated by the blue arrow.

Cardiac catheterization was performed urgently on the same day using the right common femoral artery and showed occlusion of all three native coronaries (syntax score of 62). Calcification was non-severe in all three coronary arteries and no collaterals were noted. Selective angiography of the left coronary artery revealed retrograde filling of the saphenous vein graft (SVG) through the first diagonal branch. The SVG showed a very tight stenosis at the distal anastomosis with thrombus being responsible for TIMI flow grade 2 (Figures 2A,B, Supplementary Videos 1A,B,E,F). The left internal mammary artery graft to the left anterior descending (LAD) coronary artery was found patent (Supplementary Video 1C), whereas a good long-term result after PCI and stent placement 7 years ago was observed in the vein graft to the RCA (Supplementary Video 1D). Using a 6 French (6F) Amplatz left curve (AL) guiding catheter and a SION blue guidewire (ASAHI) for lesion crossing and thrombus, aspiration was subsequently attempted using a 6F catheter (Eliminate™, Terumo); however, it was not possible to advance the thrombectomy catheter through the high-grade stenosis. An intravascular ultrasound (IVUS) catheter could also not be advanced through the lesion. In addition, since the lesion and the thrombus were located at the distal part of the bypass graft, the placement of an embolic filter protection device was not deemed technically feasible. Activated clotted time (ACT) was controlled, being at 325 s at this time point. After lesion predilatation using a 2.0×15 mm semi-compliant balloon, two DESs (3.5 mm × 16 mm and 4.0 mm × 12 mm) were deployed with short overlap. However, the remaining thrombus was noticed distally to the implanted stents (Figure 2D, Supplementary Video 1G). The implantation of another DES was deferred in the absence of residual vascular plaque and due to the risk of distal embolization with bypass graft occlusion. Instead, an intra-arterial injection of 10 mg Actilyse (recombinant tissue plasminogen activator, rt-PA; Boehringer Ingelheim, Ingelheim, Germany) was performed through the guideliner directly to the thrombus, which resulted in substantial thrombus reduction within 15 min (Figure 2E, Supplementary Video 1H).

After completion of the procedure, vascular access hemostasis was achieved by Starclose deployment. The patient was transferred to our intermediate care unit. No bleeding complications occurred. After coronary reperfusion, the patient stopped complaining of symptoms. He was put on dual platelet inhibition with aspirin once per day and ticagrelor twice per day for 12 months and additionally received fondaparinux for the next 3 days.

**Figure 3:**
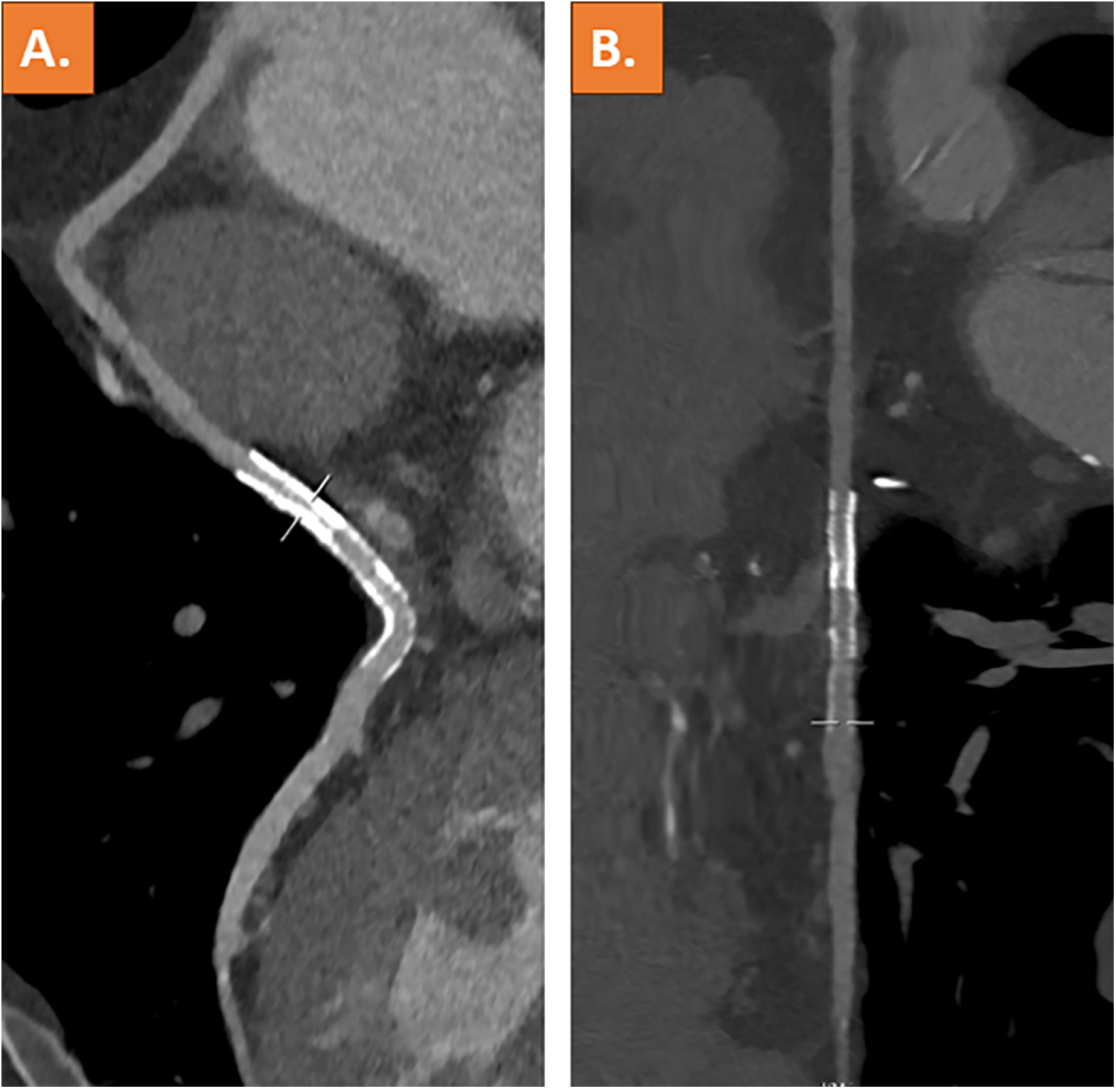
Coronary computed tomography angiography with curved multiplanar reformation **(A)** and stretched vessel view **(B)** showing a patent venous coronary artery bypass graft including stent lumen and complete resolution of the thrombus.

Coronary computed tomography angiography (CCTA), which was performed after 3 days, showed complete resolution of the thrombus and a patent vein graft (Figures 3A,B). The patient was discharged on the following day and remained on treatment with dual platelet inhibition for 12 months.

## Discussion

After CABG, patients can present with challenging lesions in the setting of an acute coronary syndrome. Degenerated SVGs typically show lesions with high thrombus burden. Techniques including thrombectomy in combination with local delivery of glycoprotein-IIb/IIIa-inhibitors have been reported for these venous graft lesions (8, 9). Because thrombectomy was unsuccessful and the thrombotic clot burden remained high despite PCI with stent placement at the proximal part of the lesion, intra-arterial rt-PA was used as a bail-out strategy in the present case. Although data on the use of catheter-directed thrombolysis is present with thrombosed peripheral bypass grafts, data on the effectiveness of local lysis in coronary bypass grafts are scarce (10). Indeed, findings of the most relevant trial data on intracoronary thrombolysis in ST-elevation myocardial infarction (STEMI) were subsumed within a recent meta-analysis by Rehan et al. (11) suggesting that it could improve major adverse cardiac events in patients undergoing primary PCI, without significantly increasing the rate of major bleeding. While in the presented case local application of rt-PA was performed at the interventionalist's discretion with the rationale that it may be more efficient than glycoprotein-IIb/IIIa (GP-IIb/IIIa) inhibitors, one can controversially discuss the comparative value of both treatment strategies. The only prospective randomized data within this context stem from a small single-center pilot study, that could not prove a reduction of infarct size by intracoronary application of tenecteplase vs. the meanwhile withdrawn GP-IIb/IIIa-inhibitor abciximab in patients with anterior STEMI undergoing primary PCI (12). Of course, these findings warrant further investigation in sufficiently powered contemporary randomized controlled trials. This evidence is necessary to determine the future role of intracoronary thrombolysis beyond a case-by-case decision. Nevertheless, local thrombolysis can be a viable option in case of no-reflow or slow-flow, which often occurs in case of SVG PCI (13). A table with principal reports on pharmacological treatment in addition to PCI of thrombotic SVG failure is provided (Table 1) (8, 9, 14–16).

**Table 1:** Principal reports on pharmacological treatment in addition to percutaneous coronary intervention of thrombotic saphenous bypass graft failure.

Author	Year	*N*	Pharmacological treatment	Route of administration
Grines et al. ( 9 )	1990	14 grafts	Fibrinolysis (urokinase, streptokinase)	Systemic (i.v.), local (catheter-directed into graft), or both
Hartmann et al. ( 14 )	1991	46 patients	Fibrinolysis (urokinase)	Local (catheter-directed into graft)
Roffi et al. ( 15 )	1992	78 patients	GP-IIb/IIIa-inhibitor (abciximab or eptifibatide)	Systemic (i.v.)
Chow and Hon ( 16 )	1996	1 patient	Vitamin K antagonist (warfarin)	Systemic (p.o.)
Vallakati et al. ( 8 )	2013	1 patient	GP-IIb/IIIa-inhibitor (abciximab)	Systemic (i.v.) and local (catheter-directed into graft)

CABG, coronary artery bypass graft; GP, glycoprotein; PCI, percutaneous coronary intervention; SVG, saphenous vein graft.

In the management of recurrent ischemia due to thrombus formation in a venous bypass graft, alternative treatment options need to be considered carefully (13). One option is PCI or even recanalization of native coronary arteries. This approach is technically challenging and may not always be immediately successful due to the complexity of the procedure and the condition of the native arteries after long-term occlusion. An additional alternative is redo-CABG, which generally is not advisable in elderly patients with a history of previous cardiac surgery.

In this case, aspiration thrombectomy was unsuccessful. Nevertheless, thrombectomy can be used in case of ectatic vessels and large thrombotic burden. The underlying concept is to remove thrombotic material from the site of the ruptured plaque to effectively restore antegrade blood flow, alleviate distal thrombus embolization during PCI, to preserve downstream microcirculation, and to avoid a no-reflow phenomenon. However, the relevance of unselective thrombectomy for patient outcomes remains controversial. Since large randomized controlled trials, such as Thrombus Aspiration in ST-Elevation Myocardial Infarction in Scandinavia (TASTE) by Fröbert et al. (17) and ThrOmbecTomy with PCI vs. PCI ALone in Patients with STEMI (TOTAL) by Jolly et al. (18), failed to improve outcomes, current guidelines do not recommend the routine use of thrombectomy (4, 19). However, a meta-analysis showed reduced cardiovascular mortality in the subgroup of patients with large thrombus burden (20), thus emphasizing the role of patient selection. Recently, dedicated mechanical thrombectomy devices were reported to overcome the limitations of manual thrombus aspiration for coronary artery use because of a larger diameter of the catheter lumen and continuous aspirating force during the procedure (21, 22). Although a novel dedicated mechanical thrombectomy device was not available in the present case, this constitutes a viable first-treatment approach. Thrombolysis could then serve as an escalation strategy to restore sufficient flow in the affected vessel with superimposed thrombus. PCI of a culprit lesion should be performed, whenever this is found to be an adequate target. Modern DESs are the gold standard for this purpose. While early generation bioresorbable vascular scaffolds have been associated with target lesion failure and scaffold thrombosis, the concept of these devices may be suited for PCI in venous grafts (23). Nevertheless, recent device improvements, such as reduced strut thickness, need to be proven effective in large randomized controlled trials before wide clinical adoption.

While utilization of embolic protection was not deemed technically feasible here, these devices could be particularly helpful when treating the culprit lesion in venous grafts due to the high risk of distal embolization of thrombotic material. Although the available study data do not support their routine use (24), embolic protection devices in conjunction with advanced interventional techniques and intravascular imaging techniques could mitigate the risk of restenosis and target vessel failure in selective clinical scenarios of vein graft PCI.

The standard of care after complex coronary intervention involves an invasive coronary angiography as a second look. However, due to thrombolysis, another invasive coronary angiography would have posed further risks and potential complications for our patient. Based on a thorough risk-benefit analysis, CCTA was chosen as a non-invasive alternative for the re-evaluation of the thrombotic burden of the venous graft. CCTA represents a particularly well-suited imaging method for assessing coronary artery bypass graft patency with excellent spatial and temporal resolution (25, 26). In addition, CCTA vs. invasive coronary angiography was recently shown to reduce procedural time and contrast-induced nephropathy (27, 28). In line with current recommendations (28, 29), CCTA may be an alternative to second-look invasive coronary angiography for the assessment of stent patency in patients with stent diameters of 3 mm and above. With the advent of CT systems offering ultra-high resolution, the future role of CCTA in these clinical scenarios could be expanded to inner stent diameters below 3 mm (30).

In addition, the role of intravascular imaging needs to be mentioned. Thus, IVUS and optical coherence tomography (OCT) are well-established methods for the characterization of lesion extent and morphology. In our case, the advancement of an IVUS catheter was unfortunately not technically feasible due to vessel tortuosity. Despite these limitations, the present case report highlights challenges of acute and recurrent ischemic events in patients with a history of extensive revascularization. In this context, the potential of a multifaceted management approach involving advanced interventional treatment in combination with local drug therapy for optimizing patient outcomes is underscored. Finally, CCTA proved to be an effective non-invasive complement as a second-look strategy for judging the effectiveness of the invasive pharmaco-chemical treatment.

### Patient perspective

The patient, who gave written informed consent for publication of this case report, was reserved toward a repeated invasive coronary angiography procedure after pharmaco-mechanical treatment of his subtotally occluded vein bypass. Indeed, after discussing the alternatives, he expressed a clear preference for a non-invasive follow-up assessment of the treatment result graft by CCTA. Our patient’s preference seems supportive of the finding of Jones et al. that the use of CCTA versus invasive coronary angiography for the assessment of CABG patency leads to improved patient satisfaction (30).

## Data Availability

The raw data supporting the conclusions of this article will be made available by the authors, without undue reservation.
