# Supplementary figures and images for: Successful pharmaco-mechanical treatment of a subtotally occluded venous bypass graft in a patient presenting with acute coronary syndrome: a case report and review of the current literature on the role of local thrombolysis
Source: Front Cardiovasc Med. 2025 Mar 17;12:1471462. doi: 10.3389/fcvm.2025.1471462 (PMC11955647; doi:10.3389/fcvm.2025.1471462)

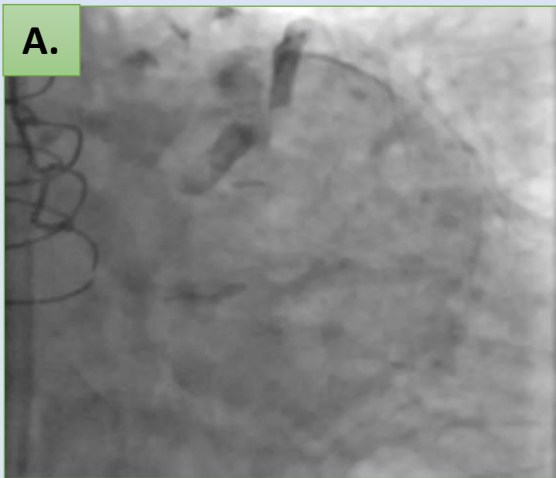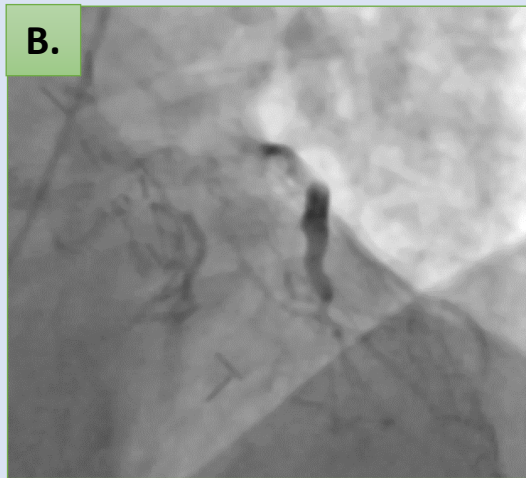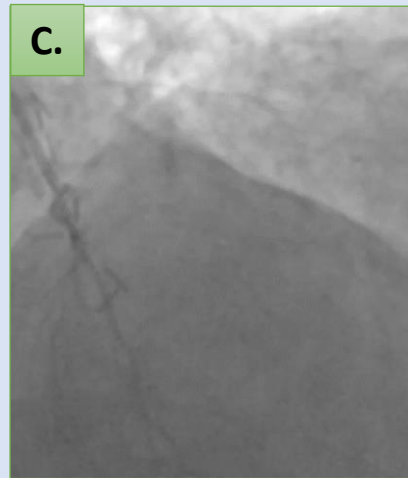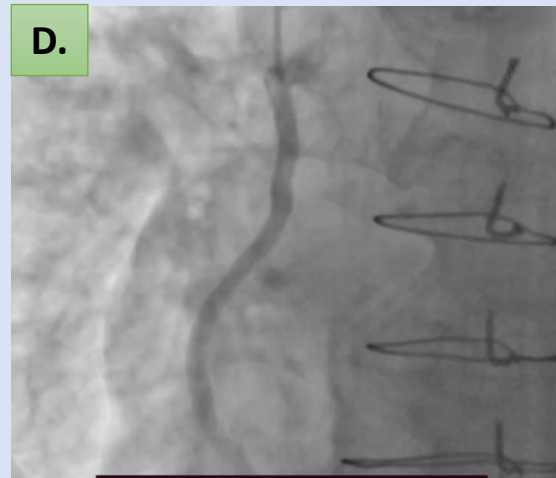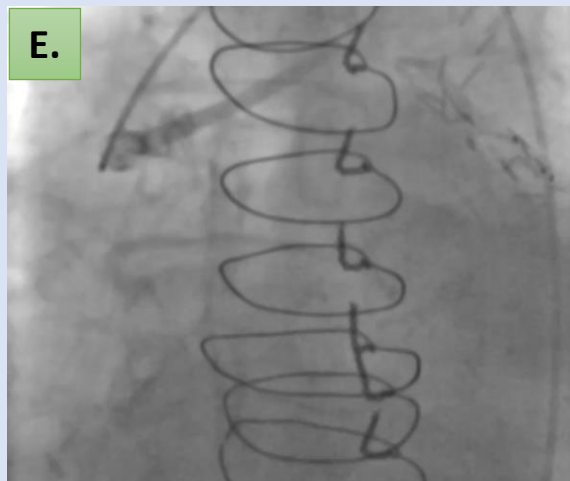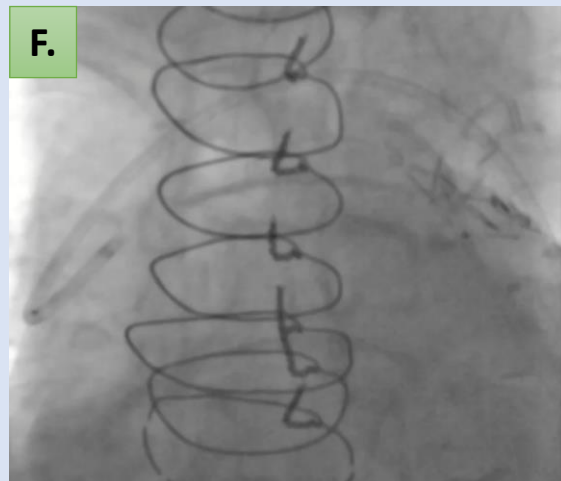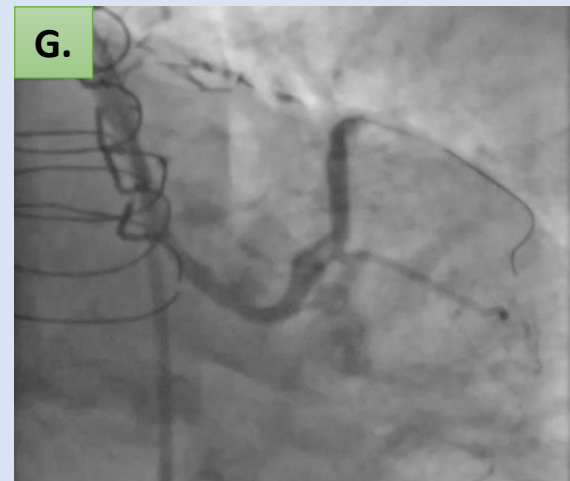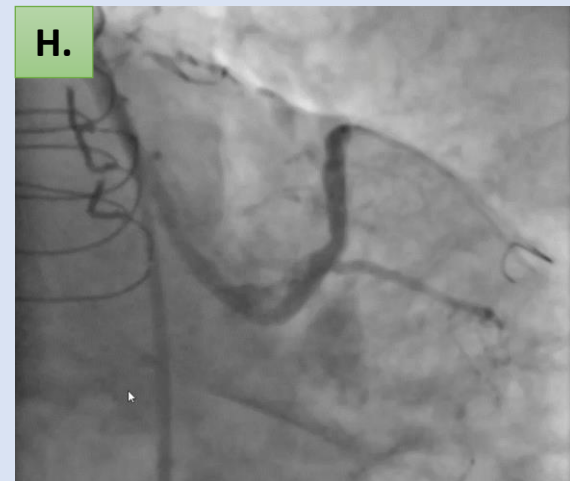

Supplement: Supplementary file 1 [file Image1.pdf]
